# Supplementary material for: Global Analysis of Natural Products Biosynthetic Diversity Encoded in Fungal Genomes
Source: J Fungi (Basel). 2024 Sep 13;10(9):653. doi: 10.3390/jof10090653 (PMC11433233; doi:10.3390/jof10090653)
Supplement: Supplementary file 1 [file jof-10-00653-s001.zip › jof-3175235-supplementary.pdf]

Supplementary Material

Global analysis of natural products biosynthetic diversity encoded in fungal genomes

Shu Zhang [1,#], Guohui Shi [1,#], Xinran Xu [1,2#], Xu Guo [1], Sijia Li [1], Zhiyuan Li [3,4], Qi Wu [1,*] and Wen-Bing Yin [1,2,*]

[1] State Key Laboratory of Mycology, Institute of Microbiology, Chinese Academy of Sciences, Beijing 100101, China
[2] Medical School, University of Chinese Academy of Sciences, Beijing 100049, China
[3] Center for Quantitative Biology, Academy for Advanced Interdisciplinary Studies, Peking University, Beijing 100871, China
[4] Peking-Tsinghua Center for Life Sciences, Academy for Advanced Interdisciplinary Studies, Peking University, Beijing 100871, China

* Correspondence: yinwb@im.ac.cn; wuqi@bimsa.cn
[#] These authors contributed equally to this work.

This PDF file includes:

Figures S1 to S9

Tables S1

Supplementary References

Supplementary Figures

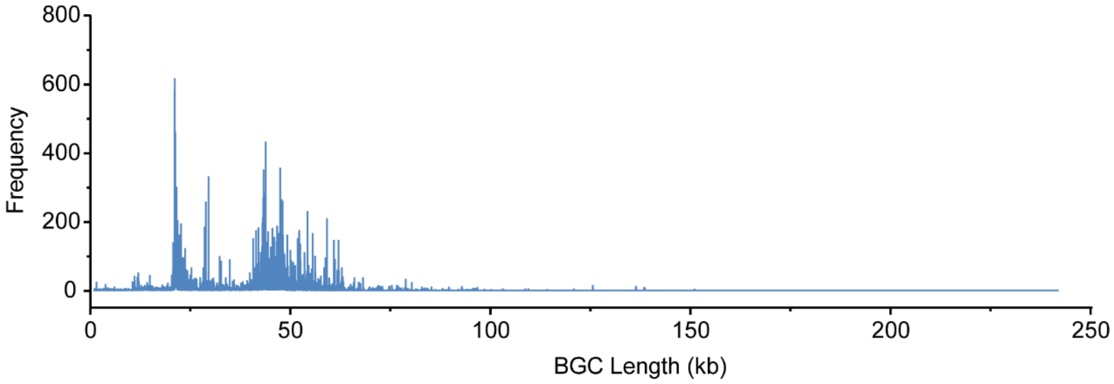

Figure S1. The length of each BGCs in the fungal kingdom. The BGCs were predicted by antiSMASH (taxon fungi, v6.1.1)[1]. The horizontal axis is the length and the vertical axis is the frequency of BGC with that length.

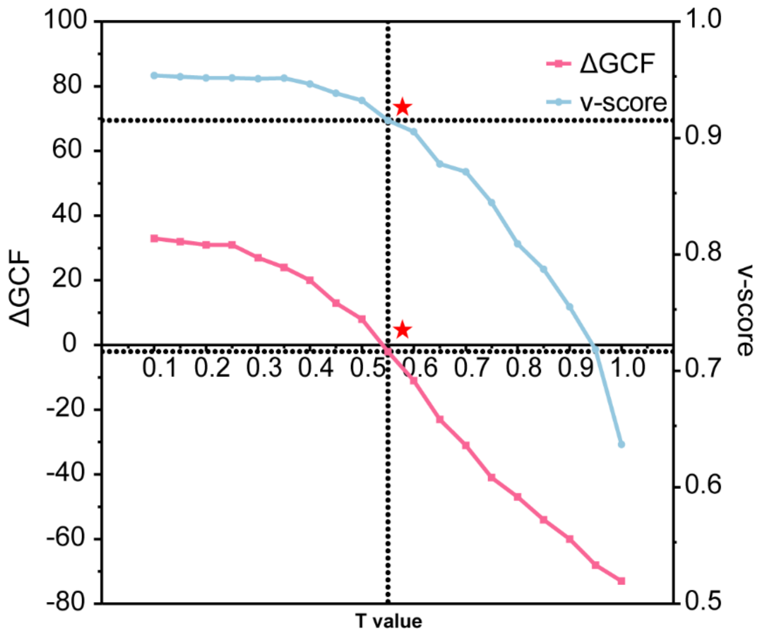

Figure S2. ΔGCF and v-score at different T-value. For the BGCs matched with MIBiG[2] in NPAtlas[3], BiG-SLiCE (v 1.1.1)[4]was utilized to select T 0.1-1.1 for clustering, and the evaluation criteria ΔGCF and v-score for clustering results at each T were compared, and it was found that V-score=0.92 and ΔGCF=-2 at T-value of 0.55 was the most appropriate.

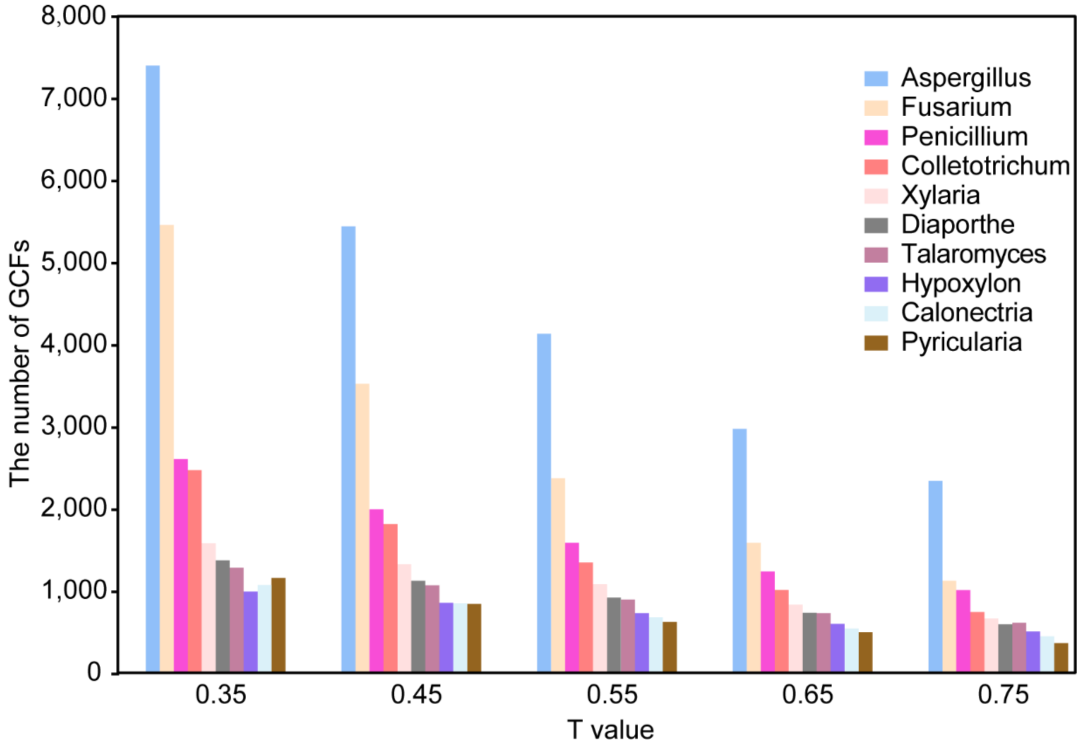

Figure S3. Genera with the highest number of GCFs at different T values. Despite the variation in the number of GCFs at different T values, the overall trend in the number rankings between genera changed little.

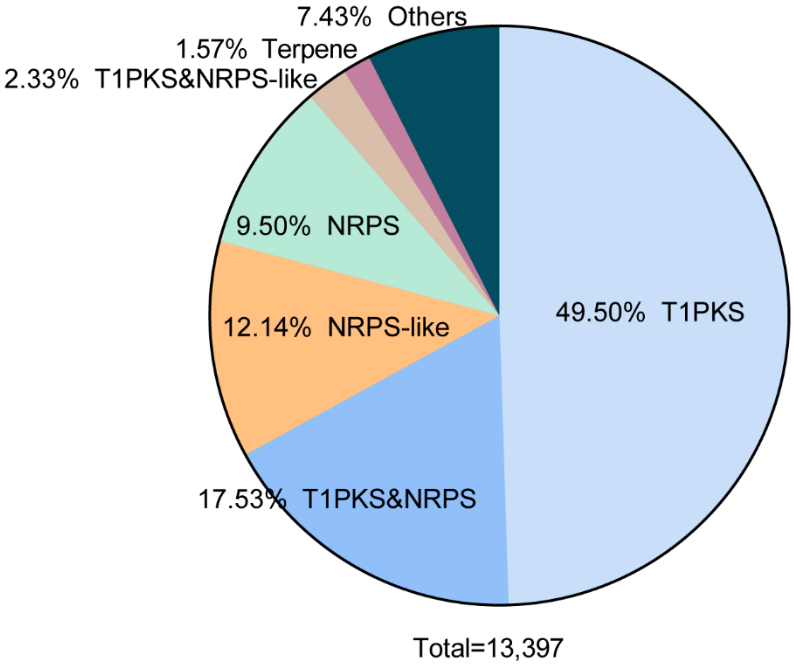

Figure S4. The type of GCFs with only 1 BGC. The GCFs were as defined by BiG-SLiCE, T = 0.4. There was a total of 13,397 GCFs with only 1 BGC, of which the number of GCFs each type in descending order, was T1PKS (49.50%), T1PKS&NRPS (17.53%), NRPS-like (12.14%), NRPS (9.50%), T1PKS&NRPS-like (2.33%), Terpene (1.57%), and the other type (7.43%).

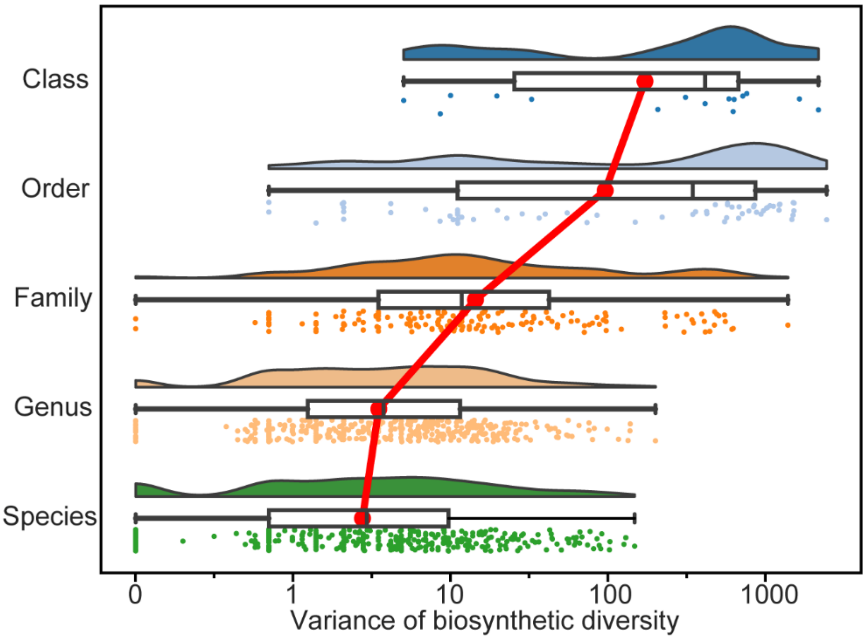

Figure S5. The variance of biosynthetic diversity of each taxon level in the fungal kingdom. Each boxplot represents the dispersion of variance values of a certain taxonomic rank, computed from the number of GCFs (defined by BiG-SLiCE[4] at T = 0.55) of the immediately lower rank. The boxplots' center line represents the median value; the box limits represent the upper and lower quartiles. Whiskers represent 1.5× interquartile range. Points outside of the whiskers are outliers. Sample sizes: Classes n = 48, Orders n = 142, Families n = 367, Genera n = 1,007, Species n = 3,011. Jittered raw data points are plotted under the boxplots for better visualization of the values' distribution. The red line connects the mean variance values of each rank. In dispersion of variance values from the order rank to the family and from the family rank to the genus, there is a significant drop in each process. From the genus rank to the species, the mean decreased insignificantly, suggesting that the number of GCFs was close between species in the same genus, while the number of GCFs varied a

lot between genera in the same family.

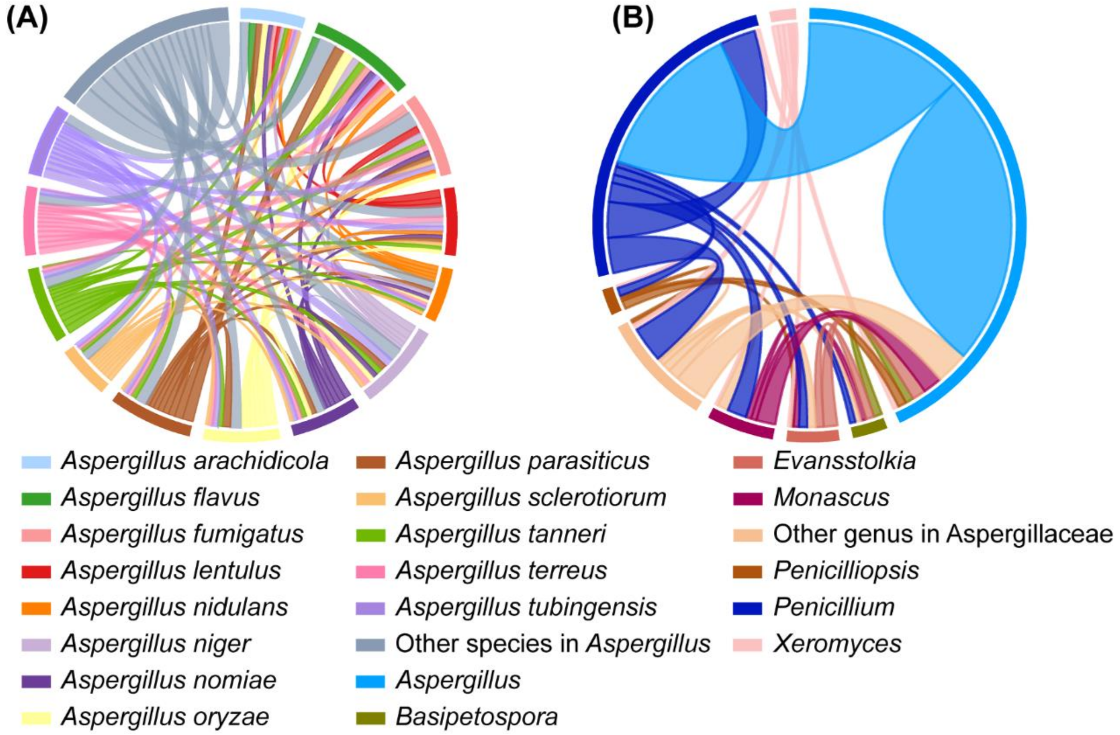

Figure S6. Distribution of GCF in each member of *Aspergillus* and Aspergillaceae.

(A) GCFs were shared among species in the genus *Aspergillus*. The band pairwise overlaps between two species were the GCFs shared in the two species. (B) GCFs shared among genus in the family Aspergillaceae. Combining (A) and (B) shows that there is a high number of *Aspergillus* genus-specific GCFs in Aspergillceae, but in *Aspergillus*, most GCFs are shared between species.

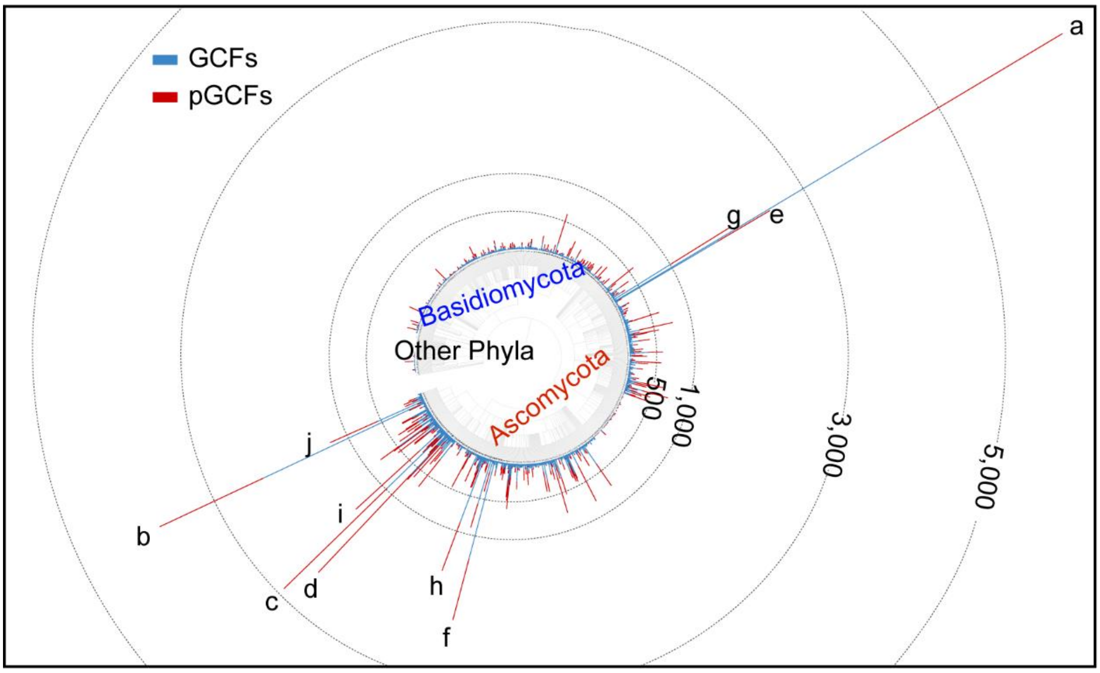

Figure S7. The number of GCFs and pGCFs at the genus level. Dilution curves for the number of pGCFs per genus. Pink marks the genera with pGCFs over 1,000, and blue marks genera with pGCFs below 1,000 (BiG-SLiCE T = 0.55). Top genera with the most potential GCFs include the following: a, *Aspergillus*; b, *Fusarium*; c, *Xylaria*; d, *Hypoxylon*; e, *Penicillium*; f, *Colletotrichum*; g, *Talaromyces*; h, *Diaporthe*; i, *Nemania*; j, *Calonectria*.

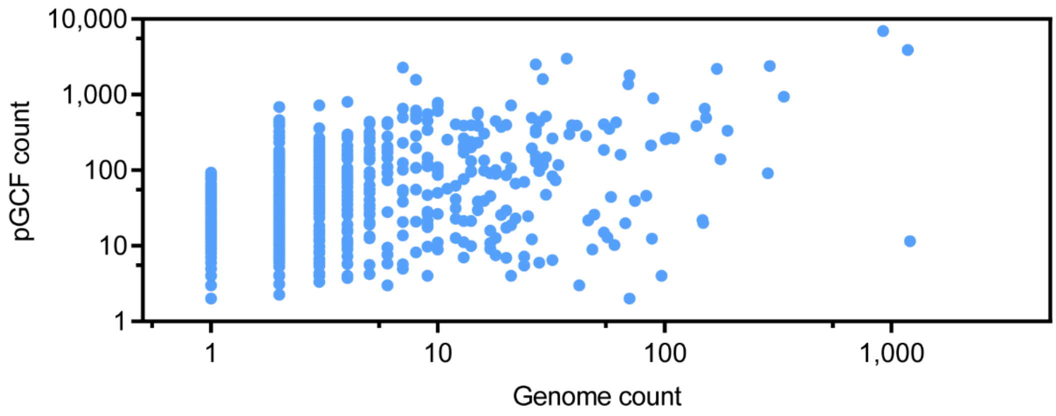

Figure S8. The number of pGCFs and genomes per genus. The X-axis indicates the number of genomes per genus, and the Y-axis indicates the pGCFs the number of pGCFs predicted when the genome count = x.

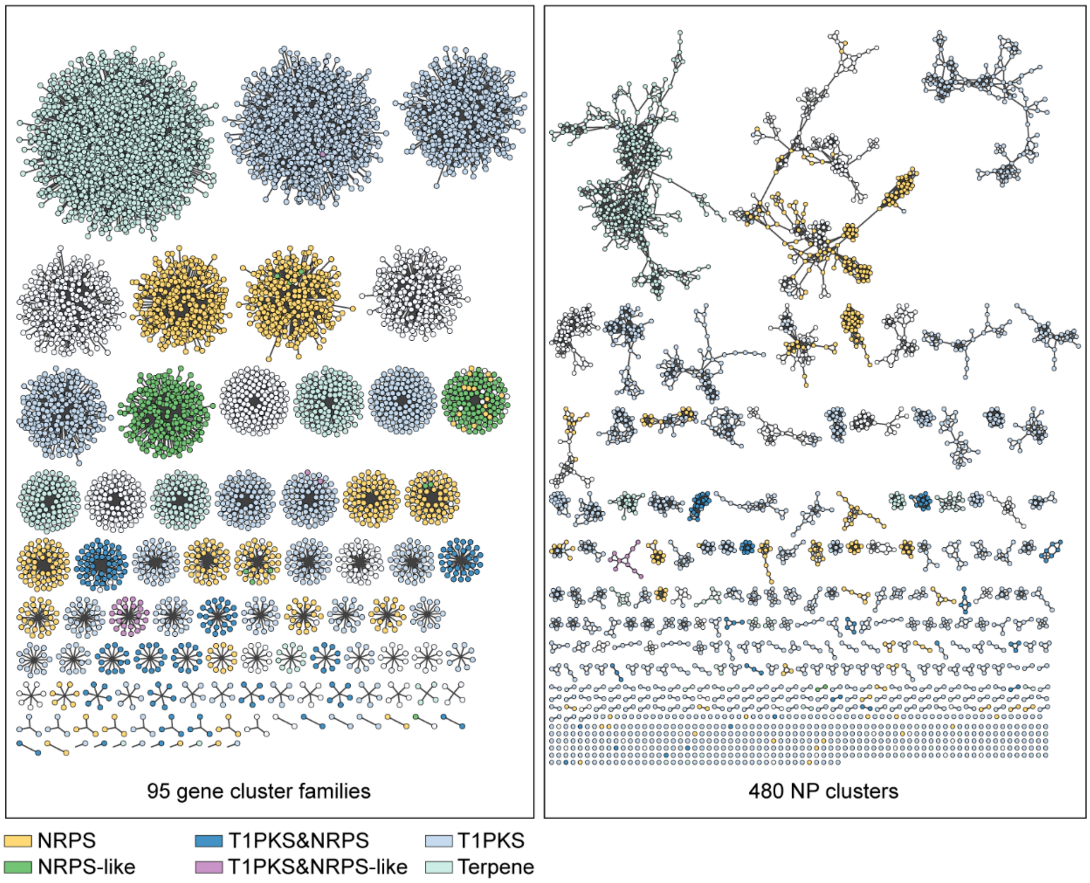

Figure S9. The GCFs containing known BGCs with corresponding compound clusters. The figure on the left shows a network of 95 GCFs, where each point represents 1 BGCs, and the distance from the node to the center represents the distance from the BGC to the GCF model, defined by BiG-SLiCE (v 1.1.1). The figure on the right shows the compounds in the same genus as the BGCs in each GCF on the left. The color of each node is the type of BGC as shown in the legend.

Supplementary Tables

Table S1 The number of GCFs at different T-value in the fungal kingdom.

Genomes	BGCs	T	GCFs
11598	293926	0.35	54708
		0.45	38171
		0.55	26825
		0.65	18092
		0.75	12971

Supplementary Reference

1. Blin, K.; Shaw, S.; Kloosterman, A. M.; Charlop-Powers, Z.; van Wezel, G. P.; Medema, Marnix H.; Weber, T., antiSMASH 6.0: improving cluster detection and comparison capabilities. *Nucleic Acids Res.* **2021,** *49* (W1), W29-W35.
2. Terlouw, B. R.; Blin, K.; Navarro-Muñoz, J. C.; Avalon, N. E.; Chevrette, M. G.; Egbert, S.; Lee, S.; Meijer, D.; Recchia, Michael J. J.; Reitz, Zachary L.; van Santen, Jeffrey A.; Selem-Mojica, N.; Tørring, T.; Zaroubi, L.; Alanjary, M.; Aleti, G.; Aguilar, C.; Al-Salihi, Suhad A. A.; Augustijn, Hannah E.; Avelar-Rivas, J A.; Avitia-Domínguez, Luis A.; Barona-Gómez, F.; Bernaldo-Agüero, J.; Bielinski, V. A.; Biermann, F.; Booth, Thomas J.; Carrion Bravo, Victor J.; Castelo-Branco, R.; Chagas, Fernanda O.; Cruz-Morales, P.; Du, C.; Duncan, Katherine R.; Gavriilidou, A.; Gayrard, D.; Gutiérrez-García, K.; Haslinger, K.; Helfrich, Eric J. N.; van der Hooft, Justin J. J.; Jati, Afif P.; Kalkreuter, E.; Kalyvas, N.; Kang, Kyo B.; Kautsar, S.; Kim, W.; Kunjapur, Aditya M.; Li, Y.-X.; Lin, G.-M.; Loureiro, C.; Louwen, Joris J. R.; Louwen, Nico L. L.; Lund, G.; Parra, J.; Philmus, B.; Pourmohsenin, B.; Pronk, Lotte J. U.; Rego, A.; Rex, Devasahayam Arokia B.; Robinson, S.; Rosas-Becerra, L R.; Roxborough, Eve T.; Schorn, Michelle A.; Scobie, Darren J.; Singh, Kumar S.; Sokolova, N.; Tang, X.; Udwary, D.; Vigneshwari, A.; Vind, K.; Vromans, Sophie P. J. M.; Waschulin, V.; Williams, Sam E.; Winter, Jaclyn M.; Witte, Thomas E.; Xie, H.; Yang, D.; Yu, J.; Zdouc, M.; Zhong, Z.; Collemare, J.; Linington, Roger G.; Weber, T.; Medema, Marnix H., MIBiG 3.0: a community-driven effort to annotate experimentally validated biosynthetic gene clusters. *Nucleic Acids Res.* **2022,** *51* (D1), D603-D610.
3. van Santen, J. A.; Poynton, E. F.; Iskakova, D.; McMann, E.; Alsup, T. A.; Clark, T. N.; Fergusson, C. H.; Fewer, D. P.; Hughes, A. H.; McCadden, C. A.; Parra, J.; Soldatou, S.; Rudolf, J. D.; Janssen, E. M.; Duncan, K. R.; Linington, R. G., The Natural Products Atlas 2.0: a database of microbially-derived natural products. *Nucleic Acids Res.* **2022,** *50* (D1), D1317-D1323.
4. Satria A. Kautsar , J. J. J. v. d. H., Dick de Ridder and Marnix; Medema, H., BiG-SLiCE: A highly scalable tool maps the diversity of 1.2 million biosynthetic gene clusters. *GigaScience* **2021,** *10* (1), giaa154.
